# Management of Gastroesophageal Reflux Disease: A Review of Medical and Surgical Management

**DOI:** 10.1155/2014/654607

**Published:** 2014-02-17

**Authors:** Nirali Shah, Sandhya Iyer

**Affiliations:** Department of General Surgery, Lokmanya Tilak Municpal Medical College and General Hospital, Sion, Mumbai 400022, India

## Abstract

*Background*. Gastroesophageal reflux disease currently accounts for the majority of esophageal pathologies. This study is an attempt to help us tackle the diagnostic and therapeutic challenges of this disease. This study specifically focuses on patients in the urban Indian setup. *Materials and Methods*. This study was a prospective interventional study carried out at a teaching public hospital in Mumbai from May 2010 to September 2012. Fifty patients diagnosed with gastroesophageal reflux disease (confirmed by endoscopy and esophageal manometry) were chosen for the study. *Results*. Fifty patients were included in the study. Twenty patients showed symptomatic improvement after three months and were thus managed conservatively, while 30 patients did not show any improvement in symptoms and were eventually operated. *Conclusion*. We suggest that all patients diagnosed to have gastroesophageal reflux disease should be subjected to 3 months of conservative management. In case of no relief of symptoms, patients need to be subjected to surgery. Laparoscopic Toupet's fundoplication is an effective and feasible surgical treatment option for such patients, associated with minimal side effects. However, the long-term effects of this form of treatment still need to be evaluated further with a larger sample size and a longer followup.

## 1. Introduction 

Gastroesophageal reflux disease was not formerly a very significant problem but its incidence has shown an absolute increase in the last 20–30 years [1]. The diagnosis of gastroesophageal reflux disease is difficult to make on clinical grounds alone and relies on investigations like upper gastrointestinal endoscopy, esophageal manometry, and 24-hour pH studies. Apart from the physical symptoms attributed to the disease, the disease also has a profound effect on the quality of life of the patient [1].

Gastroesophageal reflux disease can be managed both medically as well as surgically. With the advances in minimally invasive laparoscopic surgery for gastroesophageal reflux, there has been an increasing trend towards surgical management of reflux in order to avoid long-term dependence on medications and to give a permanent cure. Laparoscopic surgery also has its own inherent risks related to the procedure. Currently there is no clear-cut consensus about which form of treatment is suited for which patient.

This study is an attempt to help us tackle this diagnostic and therapeutic challenge of gastroesophageal reflux disease. This study specifically focuses on patients in the urban Indian setup.

## 2. Materials and Methods

This study was a prospective interventional study carried out at a teaching public hospital in Mumbai from May 2010 to September 2012 after obtaining the institute's ethics committee approval. All patients with suspected gastroesophageal reflux disease were evaluated for their symptoms and quality of life. Diagnosis of gastroesophageal reflux disease was confirmed by endoscopy and esophageal manometry. 50 such patients (with the necessary inclusion and exclusion criteria and giving written informed consent) were chosen for the study.

*Inclusion Criteria.* Newly diagnosed cases of uncomplicated gastroesophageal reflux disease with hiatus hernia patients (aged between 20 and 60 years) with symptoms of gastroesophageal reflux disease whose diagnosis has been confirmed by endoscopy and manometry.

*Exclusion Criteria.* Presence of comorbid conditions like hypertension and diabetes mellitus as well as pregnancy.

A detailed history and physical examination was done for all the patients enrolled for the study. An inquiry was made for the presence of predisposing factors—alcohol consumption, tea/coffee drinking (more than two cups/day), smoking/tobacco chewing, sedentary lifestyle, and spicy, oily, and non-vegetarian food. All patients having symptoms of gastroesophageal reflux (heartburn, regurgitation, dysphagia, angina-like chest pain, and respiratory symptoms: cough and hoarseness) had their symptoms evaluated by the visual analogue scale (scored between 1 and 10) A score was given from 1 (worst possible symptom) to 10 (no symptom) [2].

The patients were subjected to upper gastrointestinal endoscopy (to look for presence of hiatal hernia and grade of esophagitis) and high resolution esophageal manometry (to look for pressure of lower esophageal sphincter, relaxation of lower esophageal sphincter, presence of hiatal hernia, and motility of esophageal body) to confirm the diagnosis. Hiatus hernia was diagnosed when the high pressure zone produced by the lower oesophageal sphincter gastroesophageal junction was at least 2 cm higher than the high pressure zone produced by the diaphragmatic crura (double high pressure zone or double hump). Only patients showing presence of hiatal hernia on both endoscopy and manometry were included in the study.

Patients diagnosed to have gastroesophageal reflux (with the necessary inclusion and exclusion criteria) were given a trial of conservative management (lifestyle changes and medications). Lifestyle changes included eating a low-fat, bland vegetarian diet, assuming an upright (head high/propped up) position while sleeping, abstaining from tea/coffee/alcohol, and avoiding a sedentary lifestyle.

Medications included a proton-pump inhibitor (tablet Pantoprazole 40 mg twice a day) and a prokinetic agent (tablet Levosulpiride 75 mg twice a day) given for a period of three months. Patients who improved symptomatically were continued on medical management. Those patients whose symptoms did not improve with conservative management and patients who required escalating doses of medications for symptom relief were subjected to laparoscopic Toupet's fundoplication. Pneumoperitoneum was created by closed technique via a supraumbilical port. Dissection was carried out at hiatus, and fundus of stomach was mobilised and passed through a window created behind the gastroesophageal junction (shoe-shine technique). A 270° posterior wrap was performed. Fundus was sutured to oesophagus by interrupted stitches. Crural stitches were placed in case the crura were far apart and the opening was too wide. Nasogastric tube was removed on postoperative day one and sips begun. Soft diet was begun on the evening of the first postoperative day and the patient was discharged the next day in case of an uneventful recovery. Medications (proton pump inhibitors and prokinetic drugs) were continued for one month postoperatively.

All patients were followed up for a period of 9 months after diagnosis (6 months after surgery for operated patients). Outcomes after treatment were evaluated by both subjective and objective criteria.

1. Improvement in symptoms (assessed by visual analogue scale) at 3 and 9 months after diagnosis.
2. Improvement in quality of life (assessed by SF-36 questionnaire) at 3 and 9 months after diagnosis. A score was obtained for eight specific areas of functional health status—physical functioning, role limitation due to physical health, role limitation due to emotional problems, energy/fatigue, emotional well-being, social functioning, pain, and general health [2].
3. Changes in endoscopy findings at 9 months from diagnosis (6 months after surgery).
4. Changes in manometry findings at 6 months after surgery.

Patients managed surgically were also evaluated for complications: intraoperative bleeding requiring blood transfusion, diaphragmatic injury, pleural breach, splenic injury, esophageal perforation, gastric perforation, postoperative dysphagia, and wound infection.

Results were analyzed using Student's *t*-test, chi-square test, and Wilcoxon sign rank test.

## 3. Results and Discussion

Fifty patients diagnosed to have gastroesophageal reflux disease (confirmed by endoscopy and esophageal manometry) were included in the study. 20 patients showed symptomatic improvement after three months and were thus managed conservatively, while 30 patients did not show any improvement in symptoms and were eventually operated.

88% of cases were in the age group of 20–40 years while 12% cases were in the age group of 41–50 years. Mean age of patients was 32.20 years. 50% of the total cases were females in this study. Mean height, weight, and BMI of the patients were within normal limits. These findings are comparable to those found in the study by Lal et al. [3], where mean age was 37.38 years and 35.33 years for the groups treated by laparoscopic Nissen's and laparoscopic Toupet's fundoplication, respectively [3]. They had also found that gastroesophageal reflux disease had equal sex distribution (50% for males and females) [3]. Nagpal et al. [4] found that 57.14% of the patients were males and 42.86% were females [4].

72% cases had daily intake of tea or coffee (more than 2 cups per day) and 68% cases had sedentary life style, whereas 50% cases had spicy and oily food and 46% had non-vegetarian diet. 32% cases had alcohol consumption and smoking/tobacco chewing, respectively. This is in accordance with the study by Somi et al. [5], where drinking excess amount of tea was associated with symptoms of gastroesophageal reflux disease [5].

Heartburn (94%) and regurgitation (92%) were the most common symptoms at the time of diagnosis. Dysphagia (16%) was uncommon. Angina like chest pain and respiratory symptoms (cough and hoarseness) were not seen (Table 1). In the study done by Nagpal et al. [4], the most common symptom was heartburn, followed by regurgitation and constipation [4]. In a study of 107 patients done by Balsara et al. [6], the symptoms on presentation were heartburn in all (100%), regurgitation in 43 (50.59%), and volume reflux in 39 (45.88%) patients [6].

On endoscopy, hiatal hernia was present in 100% of the cases at diagnosis. All the patients had type I (sliding) hiatal hernia. Esophagitis was present in 66% patients (mainly Grade A and Grade B) at diagnosis (Table 2).

On esophageal manometry, there was a hypotensive lower esophageal sphincter with complete relaxation and presence of hiatal hernia in 100% of the cases. Esophageal body motility was normotensive in the majority of cases (88%) and was hypotensive in only 12% (Table 3). No studies have documented manometric findings in such detail.

Barium studies were not done as they are outdated now. 24-hour pH studies could not be done as they are expensive and not available in our public setup.

After three months of conservative management (with lifestyle changes, tablet Pantoprazole 40 mg twice a day, and tablet Levosulpiride 75 mg twice a day), heartburn (54%) and regurgitation (50%) were the persistent symptoms. Overall, there were 30 patients who were still symptomatic (60% cases) after three months of conservative management. This is in accordance with the findings of Sifrim and Zerbib [7], who noted that approximately a third of patients with suspected gastroesophageal reflux disease are resistant or partial responders to proton pump inhibitors [7]. These patients were subjected to surgery, that is, laparoscopic Toupet's fundoplication. Nissen's fundoplication was not preferred due to the greater incidence of postoperative dysphagia [3]. Also, most patients were poor and hailed from the rural interiors and would not be able to follow up regularly and afford repeated dilatations if required.

Transient postoperative dysphagia was the commonest complication, seen in 46.66% of the cases. However, it was only temporary and subsided within 6 weeks in all cases without any treatment, except for reassurance and adjustment of food habits. The rare complications of pleural breach, splenic injury, and esophageal perforation occurred in 1 case each and these 3 cases required conversion to open surgery. These complications occurred in the initial period of the study, demonstrating that there is a learning curve in laparoscopic surgery. Wound infection was seen in 30% of the cases; however, it was always a minor infection requiring removal of a single skin suture. None of the patients with wound infection developed fever or required incision and drainage or increase in duration of hospital stay. No patient developed a major infection that persisted for 10 or more days. This is in accordance with the study of 10 patients by Parshad et al. [8], where one patient (10%) required reexploration due to bleeding from a short gastric vessel. The most frequent postoperative complication was temporary dysphagia in 60% of patients, which improved with conservative management over 2 to 3 weeks [8].

After 3 months of medical management, mean score of heartburn showed statistically significant rise of 1.17 times (117%) in 20 patients. These patients were continued on conservative management while the other 30 were operated. At 9 months, mean score of heartburn showed significant increase of 1.50 times (150%) among the operative group and 1.30 times (130%) in the conservative group from baseline. After 3 months of medical management, mean score of regurgitation showed statistically significant increase of 1.07 times (107%) in 20 patients. Thus these patients were continued on conservative management while the other 30 patients were operated. At 9 months, mean score of regurgitation showed significant increase of 1.08 times (108%) among the operative group and 1.11 times (111%) in the conservative group from baseline. These findings were similar to those obtained in the review of four trials by Wileman et al. [9].

On endoscopy, 100.0% cases had hiatal hernia at baseline. At 3 months after surgery, 96.66% cases did not have hiatal hernia as compared to baseline. This difference was statistically significant. Overall, though 22 patients (73.33%) had esophagitis before surgery, only 1 patient had persistent esophagitis after surgery. Thus 70% patients showed improvement in esophagitisafter surgery. These findings are similar to those obtained by Parshad et al. [8], where 8 out of 9 patients (88%) had endoscopic resolution of esophagitis [8].

At 6 months after surgery, 96.6% patients showed a normotensive lower esophageal sphincter compared to all patients showing hypotensive sphincter before surgery and this change was statistically significant. There was a statistically significant increase in the distance of lower esophageal sphincter from central incisors after surgery. Lower esophageal sphincter relaxation remained complete both pre- and postoperatively in 100% cases. Hiatal hernia which was present in all cases (100%) pre-operatively was totally absent postoperatively (100%) and this difference was statistically significant. These findings were in accordance with those obtained by Wileman et al. [9]. Seven patients needed to continue the medications for three weeks after surgery to control symptoms. None of the patients required medications on a long-term basis.

Quality of life was assessed using the SF-36 questionnaire. Among the patients who were operated, the SF-36 score for quality of life did not show statistically significant change in all the eight parameters after 3 months of conservative management. The mean score for all the 8 parameters of quality of life showed improvement at 6 months after surgery. This increase was statistically significant in the areas of social functioning, pain, and general health. Among the patients who were managed conservatively, there was statistically significant improvement in score for physical functioning, role limitation due to physical health, role limitation due to emotional problems, emotional well-being, social functioning, and general health at 3 months. Mean score for these 6 parameters improved further at 9 months from diagnosis. Increase in mean score for pain was statistically insignificant at 3 months; however, it was statistically significant at 9 months (Table 4). In the review done by Wileman et al. [9], there were statistically significant improvements in the health-related quality of life at three months and one year after surgery compared to medical therapy.

## 4. Conclusion

The conclusions of our prospective study of 50 patients of gastroesophageal reflux disease can be summarized as follows.

1. In the urban Indian setup, gastroesophageal reflux disease was the most common in the age group of 20 to 40 years and both sexes were equally affected.
2. Lifestyle related factors like daily intake of tea or coffee, sedentary life style, spicy and oily food, nonvegetarian diet, alcohol consumption and smoking/tobacco chewing may be associated with gastroesophageal reflux disease.
3. Heartburn and regurgitation were the most common presenting symptoms in patients with gastroesophageal reflux disease.
4. The majority of patients with gastroesophageal reflux disease had hiatal hernia and esophagitis on endoscopy.
5. On esophageal manometry, all patients had hiatal hernia with hypotensive lower esophageal sphincter and complete relaxation of lower esophageal sphincter. The majority of the patients had normal esophageal motility. Findings of hiatal hernia on endoscopy were confirmed by manometry; therefore, endoscopy is a good method for screening for hiatal hernia.
6. After 3 months of medical management, 40% patients showed significant improvement in symptoms and quality of life and thus were continued on conservative management. The remaining 60% patients underwent surgery (laparoscopic Toupet's fundoplication).
7. Both the conservative and the operative groups of patients showed significant improvement in symptoms with treatment.
8. Endoscopy and manometry findings also showed significant improvement in the operative group.
9. Quality of life (evaluated by SF-36 score) also showed significant improvement in both groups.

Thus all patients diagnosed to have gastroesophageal reflux disease should be subjected to 3 months of conservative management. In case of no relief of symptoms, patients need to be subjected to surgery. laparoscopic Toupet's fundoplication is an effective and feasible surgical treatment option for such patients, associated with minimal side effects. However, the long-term effects of this form of treatment still need to be evaluated further with a larger sample size and a longer followup.

## Figures and Tables

**Table 1:** Symptomatology at presentation.

Symptom	Present (VAS score 1–5)	Absent (VAS > 5)
Heartburn	47 (94%)	3 (6%)
Regurgitation	46 (92%)	4 (8%)
Dysphagia	8 (16%)	42 (84%)
Angina like chest pain	0 (0%)	50 (0%)
Respiratory symptoms	0 (0%)	50 (0%)

VAS: visual analogue scale.

**Table 2:** Comparison of changes in endoscopy findings in operated cases.

Endoscopy findings	Baseline (*N* = 30)	3 months after surgery (*N* = 30)
Hiatal hernia present	30 (100%)	1 (3.33%)*
Esophagitis Grade A	10 (33.33%)	1 (3.33%)*
Esophagitis Grade B	11 (36.66%)	0 (0%)
Esophagitis Grade C	1 (3.33%)	0 (0%)
Esophagitis Grade D	0 (0%)	0 (0%)
No abnormality detected	0 (0%)	29 (96.6%)

By chi-square test;*significant.

**Table 3:** Comparison of changes in manometry findings in operated cases.

Parameter	Finding	Baseline (*N* = 30)	6 months after surgery (*N* = 30)
Pressure of lower esophageal sphincter	Hypotensive	30 (100%)	1 (33.33%)*
	Normotensive	0 (0%)	29 (96.66%)*
	Hypertensive	0 (0%)	0 (0%)
Relaxation of lower esophageal sphincter	Present	30 (100%)	30 (100%)
	Absent	0 (0%)	0 (0%)
Hiatal hernia	Present	30 (100%)	0 (0%)*
	Absent	0 (0%)	30 (100%)*
Motility of esophageal body (type of peristalsis)	Hypotensive	6 (20%)	2 (6.66%)
	Normotensive	24 (80%)	26 (86.66%)
	Hypertensive	0 (0%)	2 (6.66%)

By chi-square test; *significant.

**Table 4:** Table showing changes in mean score of quality of life in operated patients.

Parameter	Mean score (*N* = 30)		
	Baseline	3 months	9 months
Physical functioning	60.90	61.40	83.70
Role limitation due to physical health	63.60	64.00	84.30
Role limitation due to emotional problems	66.00	67.10	85.30
Energy/fatigue	71.10	71.10	86.30
Emotional well-being	67.90	68.80	84.80
Social functioning	73.60	73.70	88.70*
Pain	62.50	64.90	83.80*
General health	71.80	71.90	87.30*

By Student's *t*-test; *significant.
